# The Host’s Reply to *Candida* Biofilm

**DOI:** 10.3390/pathogens5010033

**Published:** 2016-03-18

**Authors:** Jeniel E. Nett

**Affiliations:** University of Wisconsin-Madison, Departments of Medicine, Medical Microbiology and Immunology, 5203 Microbial Sciences Building, 1550 Linden Drive, Madison, WI 53706, USA; jenett@medicine.wisc.edu; Tel.: +1-608-263-1545; Fax: +1-608-263-4464

**Keywords:** *Candida*, biofilm, host, neutrophil, model, mucosal, defense, matrix, monocyte

## Abstract

*Candida* spp. are among the most common nosocomial fungal pathogens and are notorious for their propensity toward biofilm formation. When growing on a medical device or mucosal surface, these organisms reside as communities embedded in a protective matrix, resisting host defenses. The host responds to *Candida* biofilm by depositing a variety of proteins that become incorporated into the biofilm matrix. Compared to free-floating *Candida*, leukocytes are less effective against *Candida* within a biofilm. This review highlights recent advances describing the host’s response to *Candida* biofilms using *ex vivo* and *in vivo* models of mucosal and device-associated biofilm infections.

## 1. Introduction

**Figure 1:**
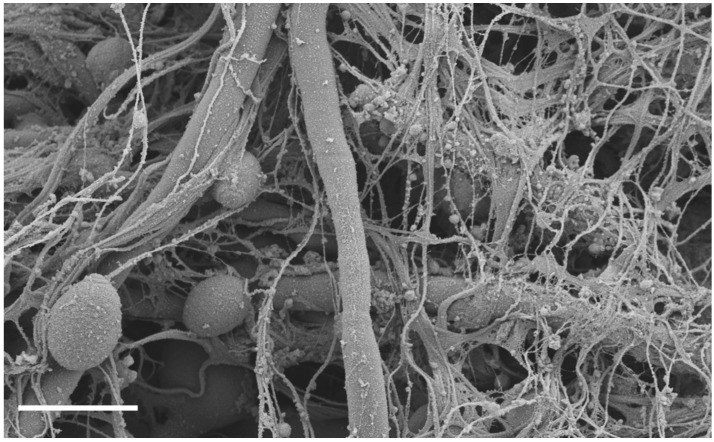
*C. albicans* biofilm infection of rat venous catheter. Following *C. albicans* instillation and a two-day growth period, catheters were processed and imaged on a JEOL 1530. Measurement bar represents 5 μm. The biofilm is composed of both yeast and hyphae embedded in an extracellular matrix of host and fungal components.

The vast majority of infections caused by *Candida* spp. involve proliferation of a biofilm on an artificial or biotic surface, such as the mucosa [1,2] (Figure 1). These adherent communities exhibit characteristics distinct from free-floating or planktonic cells, including the ability to tolerate high antifungal concentrations and to evade host immune detection [3,4,5,6]. Candidiasis is prominent in the hospital setting, with *Candida* spp. accounting for the 4th most common cause of bloodstream infection and the 3rd most common cause of urinary tract infection [7,8,9,10]. As medical care advances and device use increases, biofilm-associated infections have increased in parallel [11]. The most frequently used and infected devices include vascular catheters, urinary catheters, and dentures. However, pacemakers, artificial heart valves, voice prostheses, and central nervous system shunts are also at risk for infection [11,12]. The mortality due to invasive candidiasis, such as central venous catheter-associated infection, is astonishingly high, estimated at 26%–38% [11]. In addition, increased mortality has been observed when infected vascular catheters are retained, presumably due to the resilient nature of the biofilm communities [13].

Similar to device-associated infections, *Candida* spp. exhibit biofilm characteristics while adherent to biotic surfaces, such as the mucosa or the endothelium [14,15,16]. Mucosal biofilms are prevalent in the community. One of the most common mucosal biofilm infections, vaginal candidiasis, affects 30%–50% of women, with a subset of close to 6%–9% developing recurrent disease [17]. Oral candidiasis is similarly prevalent, particularly among patients who are elderly, immunosuppressed, or receiving antibiotics [18].

Like biofilms on abiotic surfaces, mucosal biofilms are also composed of collections of yeast and hyphal cells encased in an extracellular matrix [14,15,16,19]. However, their substrate for biofilm formation is a living structure, the mucosa, which responds to fungal adherence, secreted microbial products, and tissue invasion. Although many aspects of device-associated and mucosal biofilms are similar, the mucosal biofilms lack the adjacent abiotic surface and instead, are under the influence of immune factors induced by the *Candida*-epithelium interface [20,21]. Clinical studies demonstrating a difference in host susceptibility to *Candida* biofilm-associated infections suggest distinct immune responses to mucosal and device-associated biofilms [22]. For example, oral candidiasis is common in patients with dampened cell-mediated immunity, such as those with HIV and AIDS [18]. In contrast, denture stomatitis, a device-associated infection involving the same niche, is common in immunocompetent patients [23]. Clinical biofilms may be both mucosal and device-associated, such as dental stomatitis with oral candidiasis or vaginitis with intrauterine device infection [24,25].

**Figure 2:**
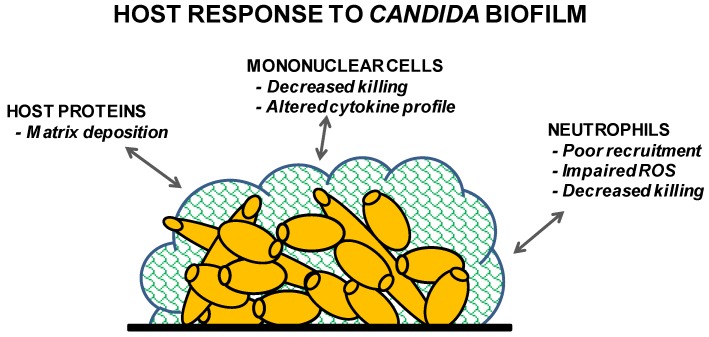
Summary of the host’s response to *Candida* biofilm.

Studies suggest that the biofilm lifestyle protects fungi from host recognition [26,27,28,29]. This is consistent with clinical studies showing that device-associated *Candida* biofilms are extraordinary difficult to cure, even for patients without immunocompromise [13,30,31]. This review will focus on how the host responds to *Candida* biofilm formation for various niches important for clinical infection (Figure 2). Although there are common themes to how the host responds to biofilms at these infection sites, variation exists. This is likely related to the differences in nutrients, host proteins, immunity, and physiological flow conditions. Most investigations have been undertaken with *C. albicans*. However, the majority of other pathogenic *Candida* spp., including *C. dubliniensis*, *C. glabrata*, *C. krusei*, *C. tropicalis* and *C. parapsilosis*, have also been shown to form biofilms of clinical significance [32].

## 2. *Ex Vivo* Models

### 2.1. Mononuclear cells

Investigations examining the leukocyte response to *Candida* biofilms have consistently shown a diminished response to these adherent communities [26,27,28,29]. Chandra *et al.* first used a co-culture system to analyze the interaction of peripheral blood mononuclear cells with *C. albicans* biofilms and identified several differences in leukocyte response [33]. When compared to co-culture with planktonic cells, mononuclear cells exposed to biofilms produced an altered cytokine profile with higher levels of IL-1β, IL-10, and MCP-1 and lower levels of IL-6 and MIP1β. As this pattern involves alteration of both pro- and anti-inflammatory pathways, it was hypothesized that mononuclear cell recognition of biofilms involves multiple interactions. Differences in phagocytic response were also observed. While the mononuclear cells elicited a phagocytic response to the planktonic cells, they migrated throughout biofilm structures without induction of phagocytosis or killing of the biofilm. Furthermore, the mononuclear cells augmented biofilm proliferation, increasing the biofilm thickness over two-fold [33]. The responsible factor has not been identified but was shown to be a soluble factor secreted into the supernatant during biofilm and mononuclear cell co-culture.

Additional investigation of the monocyte response to *C. albicans* biofilms by Katragkou *et al.* confirmed distinct differences in reaction to biofilm and planktonic cells [28]. For all conditions tested, monocytes had significantly less anti-biofilm activity. Monocyte activity (measured by damage to *Candida* in a tetrazolium salt XTT assay) against *C. albicans* biofilms was approximately half that observed for planktonic cultures. Consistent with prior investigation, the monocytes did not surround or engulf the biofilms and appeared inactive within the biofilm. Using a monocyte cell line, the authors showed a decreased pro-inflammatory cytokine response to biofilm, with reduced TNF-α release. Taken together, these *ex vivo* studies demonstrate altered recognition of *Candida* in the biofilm state. The mechanism of this is unclear but may involve masking of glucan as echinocandin treatment augmented the anti-biofilm activity of monocytes [28,34]. Surprisingly, studies examining that response to *C. parapsilosis* did not reveal significant differences in the activity of a monocyte cell line against biofilm and planktonic cells [26]. Possible etiologies underlying this phenomenon include the differences in biofilm architecture, filamentation, or extracellular matrix composition between *C. albicans* and *C. parapsilosis* biofilms.

### 2.2. Polymorphonuclear (PMN) Cells

PMNs cells exhibit diminished activity against *C. albicans* biofilms when compared to their impact on planktonic cultures [28]. In co-culture experiments, PMNs were approximately 50% less effective against biofilms, as measured by XTT. Interestingly, this difference was tightly linked to the biofilm architecture. When biofilms were physically disrupted by scraping, PMN activity increased to levels observed for planktonic cells. Further investigation questioned if cytokine-priming of PMNs or opsonization would augment the anti-biofilm activity [27]. However, pre-treatment of PMNs with interferon-γ (INF-γ) or granulocyte colony-stimulating factor (G-CSF) did not significantly enhance their activity against opsonized or unopsonized *C. albicans* biofilms. One possibility is that PMNs do not recognize *C. albicans* within a biofilm; hence, enhancement of ligand interactions by cytokine priming has minimal impact of PMN activity. This phenotype of neutrophil evasion is less pronounced for *C. parapsilosis* biofilms [26]. PMNs display similar activities against biofilm and planktonic *C. parapsilosis.*

Xie *et al.* further explored the neutrophil response to *C. albicans* biofilms to uncover the mechanism underlying the resistance to killing [29]. Several key observations were found. Compared to early biofilms (3 h), mature *Candida* biofilms (24 h) did not trigger production of reactive oxygen species (ROS) in neutrophils. The biofilm-exposed neutrophils remained viable for several hours and were able to be activated by alternative stimuli to induce fungal damage. Similar to a prior investigation, disruption of biofilm architecture promoted neutrophil activity and this was linked to increased ROS production [28]. The authors further correlated the hindered neutrophil response to the extracellular biofilm matrix, specifically, the presence of β-glucans.

## 3. Mucosal Biofilms Models

### 3.1. Oral Biofilms

Using a murine model of oral candidiasis, Dongari-Bagtzoglou *et al.* characterized *C. albicans* biofilm growth and examined the host response [16]. *Candida* biofilm induced a hyperkeratotic response and epithelial cell desquamation. Immuno-fluorescent imaging demonstrated incorporation of keratin and desquamated cells into the extracellular material surrounding the biofilm. In addition, the oral biofilm elicited neutrophil migration. Aggregates of neutrophils aligned adjacent to the biofilm with a subset migrating deeper in the biofilm. Although the neutrophils were present in the biofilm, they were not effective in clearing the infection. In part, this resistance of *C. albicans* oral biofilm to neutrophil killing appears to be due to the glycosylphosphatidylinositol (GPI)-anchored cell wall protein Hyr1 [35]. Expression of this hyphal-specific protein was found to promote resistance to neutrophil killing *in vivo* and in an *ex vivo* oral candidiasis model. Numerous investigations have examined the immune response to oral candidiasis [36,37]. Although these studies have not specifically described oral biofilms, the immune pathways involved in oral candidiasis likely apply to mucosal biofilms as well. Excellent reviews describe this response, which includes epithelial cell activation, priming of Th17 cells, and induction of cytokines, including IL-17 and IL-23 [36,37,38].

### 3.2. Vaginal Biofilms

Recent investigations have identified mucosal biofilm formation in a murine model of vaginal candidiasis [14,39,40]. Few studies have described the host response in the context of vaginal biofilms. However, considering the similarity of models used, much of current understanding of the immune response to vaginal candidiasis likely applies to vaginal *Candida* biofilms and the studies have been reviewed in more detail elsewhere [36,41,42,43]. Clinical studies demonstrate an inflammatory response with neutrophil infiltration in patients with symptomatic vaginal candidiasis [39]. However, the neutrophils appear to promote inflammation and tissue damage more than assist with fungal eradication. Using a murine vaginal candidiasis biofilm model, Yano *et al.* showed a similar pattern of neutrophil infiltration into vaginal lavage fluid [44]. In part, this response involves epithelial cell release of chemotactic factors, including alarmins S100-A8 and S100-A9 [44,45]. Although it is interesting to propose a role for IL-17 in vaginal candidiasis, a clear link has not been established [36,41]. Animal models of vaginal candidiasis demonstrate the importance of adaptive immunity [42,46]. Studies show the induction of protective immunity through vaccination and clinical trials are currently investigating the potential for use of vaccines.

## 4. Device-Associated Biofilms

### 4.1. Vascular Catheter Biofilms

Several animal models have been developed to mimic vascular catheter-associated *Candida* biofilm infection [47,48,49]. In these rabbit, rat, and mouse models, biofilms form on the surface of jugular venous catheters following luminal inoculation. Similar to mucosal biofilms, imaging of these device-associated biofilms revealed the incorporation of host cells within the biofilm [47]. While the majority of the cells were of fungal origin, few were larger and had the appearance of leukocytes. Further examination of these cells confirmed the presence of biofilm-associated neutrophils [50]. However, consistent with electron microscopy imaging, the neutrophils were relatively scarce, approximately one per 75 *C. albicans* cells. This is contrast to studies of mucosal biofilms, where neutrophils were observed to migrate throughout the biofilm [16].

A striking finding from both the rabbit and rat venous catheter biofilms was the presence of a robust extracellular matrix, more extensive than that observed for many *in vitro* conditions [47,48,50]. This observation suggested the incorporation of host proteins in the extracellular matrix and prompted proteomic analysis of the material [50]. Surprisingly, nearly all (98%) of the extracellular matrix proteins were of host origin. Over 100 host proteins of a variety of types were identified. The most abundant included hemoglobin, albumin, and alpha globulins. Several represented categories included matricellular proteins, inflammatory or leukocyte-associated proteins, and erythrocyte or heme-associated proteins. Although the role of many of these proteins is unclear, they may be involved in the immune response, fungal acquisition of iron, or scaffolding of the matrix [45,51,52,53,54,55,56]. Others may involve non-specific interactions.

### 4.2. Denture Biofilms

Several animal models have been utilized to study the host response to denture stomatitis biofilms [22,57,58,59]. For the most part, these studies have examined the inflammatory response of the adjacent mucosal tissue. Johnson *et al.* developed a rat model of chronic denture dermatitis, which utilizes custom fitted intraoral devices that can be removed and sampled over time [22,59]. Over the course of eight weeks, animals progressively developed palatal inflammation, erythema, and edema in response to the device-associated *C. albicans* biofilm [22]. Histopathology revealed prominent inflammatory infiltrates by 6–8 weeks. The palatal inflammation and lesions mimicked clinical denture stomatitis. The findings are also in line with prior investigations of chronic denture stomatitis in animal models that did not specifically examine biofilm formation [60,61].

A rat model of more acute denture stomatitis has also been utilized to examine the host response to *C. albicans* biofilm infection [50,58]. In this model, palatal devices are constructed *in situ* and device-associated biofilms form over two days. In contrast to the chronic stomatitis models, animals are immunosuppressed with corticosteroids. Similar to models of chronic infection, mucosal inflammation with leukocyte infiltration was observed [58]. However, hyphal invasion was noted as well, so it is unclear if the leukocyte infiltration was prompted by denture-associated biofilm, oral candidiasis, or the combination. Further examination of the host cells associating directly with the device revealed a combination of leukocytes and epithelial cells [50]. Host proteins were prominent in the extracellular matrix, with 132 identified. The most prevalent included amylase, hemoglobin, and antimicrobial peptides (bactericidal permeability-increasing or BPI-fold containing proteins). These findings show a variety of host cells and proteins are in direct contact with denture biofilms. Further investigations are needed to determine the role of these host components.

### 4.3. Urinary Catheter Biofilms

Wang *et al.* developed a murine model of urinary catheter-associated candidiasis and characterized the host response to biofilm [62]. In this model, catheter segments are surgically placed in the bladder and inoculated by intravesicular injection. *C. albicans* biofilm was found to illicit an inflammatory response marked by pyuria and submucosal bladder inflammation. Furthermore, their studies support a role for lysozyme, an innate immunity effector present on the mucosa and expressed by neutrophils, in the clearance of infection. Compared to the parent strains, lysozyme M-deficient mice (lysM-/-) developed higher fungal burdens and more pronounced pyuria. The inflammatory response is likely a result of not only the biofilm but also the concurrent cystitis.

The host response to *Candida* urinary catheter biofilm was further explored using a rat model [63]. In contrast to the murine model, this involves luminal inoculation of a urethral catheter. Over the course of 48 h, biofilms formed on both the luminal surface and the bladder epithelial surface. Histopathology demonstrated findings of acute cystitis, including fungal invasion and neutrophil infiltration. Using this infection model, a proteomic analysis of the extracellular material associating with the *C. albicans* urinary catheter biofilm was undertaken [50]. Analysis revealed numerous (>200) host proteins within the *Candida* biofilm. The most abundant proteins were fibrinogen, keratin, and hemoglobin. Similar to other models of infection, leukocyte-associated and inflammatory proteins were identified as well. Imaging of the biofilm individual cells confirmed the presence of few neutrophils associating with the biofilm.

### 4.4. Subcutaneous Biofilms

Several subcutaneous implant animal models have shed light on the host response to *Candida* biofilm [64,65,66]. Riciova *et al.* developed a rat model of *Candida* biofilm infection which involves implantation of vascular catheter segments in the subcutaneous tissue. Mature biofilms formed over the course of six days [64]. The animals rapidly cleared the *C. albicans* infection unless they were immunosuppressed with glucocorticoid. The robust host inflammatory response may have been related to a foreign body reaction or the adherent *C. albicans*, which had not yet formed a mature biofilm. Similarly, Nieminen *et al.* observed an inflammatory response to *C. albicans* biofilms using a murine model involving subcutaneous implantation of a chamber [66]. In addition to the wound healing response that was observed for the chamber-only (uninfected) controls, an infiltrate of neutrophils was also seen in animals with *Candida* biofilm infections. The authors further described an anti-inflammatory compound, leucine derivative DL-2-hydroxyisocaproic acid (HICA), which modulated this activity. Treatment with HICA, which is active against *C. albicans* biofilms, decreased infiltration of neutrophils and formation of granulation tissue.

## 5. Other Models

*Galleria mellonella*. Although mammalian models of biofilm-associated infection closely mimic clinical disease, there is interest in alternative invertebrate models to provide higher throughput screening, limit cost, and utilize less sentient animals. *G. mellonella* (Lepidoptera: the greater wax moth) offers an alternative model for biofilm study [67]. Although insect models lack factors for acquired immunity, they do have a well-developed innate immune system comprised of phagocytes (hemocytes of the hemolymph) and humoral components. In addition, the *G. mellonella* larvae model allows growth of *Candida* at 37 °C and collection of tissues for histology. Using this model, Borghi, *et al.* identified a correlation between *in vitro C. albicans* biofilm formation and pathogenesis [67]. On histology, invasive fungal mats resembling biofilms were visualized. Regardless of the burden, the host response included melanization and fat body cell sequestration, processes that contain infecting pathogens to the hemolymph. At the higher burden associated with more robust biofilm production, necrosis of the fat body was observed. Further studies have used optimized larval processing and histology to quantitate these associating hemocytes as well as measure the expression and activity of the host antifungal peptides and enzymes [68,69]. The unique model is a tool to examine the innate immune response to biofilm infection, although findings may be limited by differences between mammalian and invertebrate immunity.

## 6. Conclusions

The majority of *Candida* infections involve the production of surface-associated biofilm communities. Recent studies show that these structures evade host responses, including killing by both mononuclear and polymorphonuclear leukocytes. This appears to be a multifactorial process and likely varies by clinical niche of infection. Studies suggest a role for the extracellular matrix in this immune evasion phenomenon. However, much remains unknown regarding how the immune system recognizes the extracellular matrix and how many biofilms go virtually unrecognized. Novel strategies to treat fungal biofilm infections are of great interest. Further research in this area may identify biofilm-specific drug targets, including agents designed to disrupt extracellular matrix, augment current antifungal therapies, or disarm biofilm immune evasion.
